# Proximal and extended aortic arch replacement in acute DeBakey type I aortic dissection

**DOI:** 10.3389/fsurg.2023.1081167

**Published:** 2023-02-13

**Authors:** Masahiko Narita, Masahiro Tsutsui, Ryouhei Ushioda, Yuta Kikuchi, Tomonori Shirasaka, Natsuya Ishikawa, Hiroyuki Kamiya

**Affiliations:** Department of Cardiac Surgery, Asahikawa Medical University, Asahikawa, Japan

**Keywords:** acute aortic dissection, aortic repair, aortic arch, outcome, replacement range

## Abstract

**Objective:**

This study aimed to compare the short- and long-term outcomes of proximal repair vs. extensive arch surgery for acute DeBakey type I aortic dissection.

**Subjects:**

From April 2014 to September 2020, 121 consecutive patients with acute type A dissection were surgically treated at our institute. Of these patients, 92 had dissections extending beyond the ascending aorta.

**Methods:**

Of the 92 patients, 58 underwent proximal repair, including aortic root and/or hemiarch replacement, and 34 underwent extended repair, including partial and total arch replacement. Perioperative variables and early and late postoperative results were statistically analyzed.

**Results:**

The duration of surgery, cardiopulmonary bypass, and circulatory arrest was significantly shorter in the proximal repair group (*p *< 0.01). The overall operative mortality rate was 10.3% in the proximal repair group and 14.7% in the extended repair group (*p *= 0.379). The mean follow-up period was 31.1 ± 26.7 months in the proximal repair group and 35.3 ± 26.8 months in the extended repair group. During follow-up, the cumulative survival and freedom from reintervention rates at 5 years were 66.4% and 92.9% in the proximal repair group, and 76.1% and 72.6% in the extended repair group, respectively (*p *= 0.515 and *p *= 0.134).

**Conclusions:**

No significant differences were found in the rates of long-term cumulative survival and freedom from aortic reintervention between the two surgical strategies. These findings suggest limited aortic resection achieves acceptable patient outcomes.

## Introduction

With improvements in the perioperative and postoperative management of acute type A aortic dissection (AADA), there has been a trend towards aggressive total arch replacement (TAR) rather than hemi-arch replacement to improve long-term outcomes, especially in DeBakey type I aortic dissection (1–5). However, it remains unclear whether to perform extended arch repair or limited hemi-arch replacement in patients with DeBakey type I AADA because surgical volume, surgical experience, and indications for extended arch repair vary among institutions (6–9). Therefore, clinical experiences regarding this question should be reported not only by large centers of excellence, but also by smaller centers, in order to better address this question in the real-world setting.

Our institute is in the northern Hokkaido and performs approximately 20 surgeries for AADA annually. At our institute, replacement of the ascending aorta with resection of the primary entry is the preferred surgical approach. TAR is performed only in cases of (1) an intimal tear localized along the greater curvature of the aortic arch, (2) malperfusion of the carotid arteries, (3) the critical stenosis of the true lumen, and/or (4) significant dilation of the aortic arch (exceeded diameter of 50 mm), and/or (5) patient age less than 60 years. If patients are not candidates for TAR, such as in cases of old age or preoperative patient status, hemi-arch replacement is performed to minimize the invasiveness of the surgery and maximize survival rates. Thus, the aim of the current study was to compare the short- and long-term outcomes of aortic arch surgery using our two surgical strategies in patients with DeBakey type I AADA.

## Material and methods

### Ethical considerations

This study was approved by our institutional review board, which waived the requirement for informed patient consent, owing to the retrospective nature of the current study (IRB number: 19207).

### Patients

From April 2014 to September 2020, 121 adult patients with acute aortic dissection underwent urgent surgery at Asahikawa Medical University Hospital. Of these, 29 patients who underwent hemiarch replacement for DeBakey type II aortic dissection were excluded from this study. Of the 92 included patients, 58 underwent hemiarch replacement (proximal repair, PR group), whereas 34 underwent partial or total replacement (extensive repair, ER group).

### Surgical management

The operation was performed in the supine position. Median sternotomy was performed and cardiopulmonary bypass was established with arterial cannulation into the ascending aorta, femoral artery, or axillary artery determined by patient status, and bicaval venous drainage. A vent tube was inserted into the left ventricle *via* the right superior pulmonary vein. The patient was cooled to the target rectal temperature of 26 °C. During systemic cooling, the ascending aorta was cross-clamped, and cardioplegic arrest was induced in the absence of a massive thrombus in the false lumen of the ascending aorta. Otherwise, cross-clamping was avoided and cardioplegia was administered after commencing hypothermic circulatory arrest. If the ascending aorta was cross-clamped, the proximal anastomosis was performed using a modified “turn up” technique. If cross-clamping of the ascending aorta was not performed, the distal anastomosis was completed first, and the proximal anastomosis was then completed with a separate graft using the modified “turn up” technique. Concomitant procedures, such as root replacement or coronary artery bypass grafting, were performed during cardiac arrest after the completion of the distal anastomosis.

Moderate hypothermic circulatory arrest with a rectal temperature of 26°C and antegrade selective cerebral perfusion are preferred for brain protection at our institute. In cases of hemi-arch replacement, a 1-branched prosthesis was used for the distal anastomosis. In cases of extended arch repair, a 4-branched prosthesis was used, and the arch vessels were reconstructed after commencing systemic perfusion during the re-warming phase.

For TAR, a frozen elephant trunk (FET; Frozenix, Lifeline Japan, Japan) was used in most cases. In cases of a FET, the subclavian artery was reconstructed extra-anatomically using an 8 mm expanded polytetrafluoroethylene prosthesis (Gore Propaten; W Gore & Associates, Flagstaff, Ariz, United States) anastomosed to the left axillary artery through a separate incision. With this extra-anatomical procedure, all anastomoses can be performed in relatively safe position by avoiding deep distal anastomosis and avoiding in-situ reconstruction of the left subclavian artery, therefore we preferred it (10). If a FET was not used, the distal anastomosis was performed using the classical elephant trunk technique.

### Follow-up

Follow-up data on survival, the need for aortic reoperation, and the causes of death were determined from clinical records at our outpatient clinic or direct telephone interviews with patients or relatives. All required follow-up data were collected, and no patients were lost to follow-up. The mean duration of follow-up was 23.5 months (range 6.3–57.3) in the PR group and 32.5 months (range 27.8–60.5) in the ER group.

### Statistical analysis

Continuous variables were presented as means and standard deviations or medians and first and third quartiles in cases of a skewed data distribution. Categorical variables were presented as frequencies and percentages. For comparison of continuous variables, the Student's t-test was applied for normal distributions, as verified using the Kolmogorov–Smirnov test. For non-normal distributions, the Mann–Whitney rank sum test was used. Categorical variables were compared using the chi-square test. Fisher's exact test was used for small sample sizes (*n* < 5). Survival and freedom from reintervention were analyzed using the Kaplan–Meier method and log-rank calculations. All statistical calculations were performed using IBM SPSS Statistics for Windows, version 22.0. (IBM, Armonk, NY, United States). The significance level was set at *p* < 0.05.

## Results

### Indications for proximal and extended arch repair

Of the 34 patients in the ER group, 27 met our institutional inclusion criteria for TAR, which were as follows: (1) an intimal tear localized along the greater curvature of the aortic arch, (2) malperfusion of the carotid arteries, (3) the critical stenosis of the true lumen, (4) significant dilation of the aortic arch (exceeded diameter of 50 mm), and/or (5) age under 60 years. The decision to perform extended arch repair was made intraoperatively for seven patients, despite not meeting inclusion criteria. Of the 58 patients in the PR group, 10 patients met the inclusion criteria, but two of these patients required preoperative cardiopulmonary resuscitation (CPR) and two patients experienced profound shock. These four patients therefore underwent hemi-arch replacement instead.

### Preoperative characteristics

A comparison of the preoperative data between the 2 groups is shown in Table 1. Age was significantly more advanced in the PR group compared with the ER group. Patients in the ER group were more likely to have malperfusion (15.5% vs. 35.3%; *p* = 0.029). No significant differences in other preoperative factors were observed between the two groups.

**Table 1:** Preoperative status of patients.

	Overall (*n* = 92)	Proximal repair group (*n* = 58)	Extensive repair group (*n* = 34)	*p*-value
Age (years old)	72.0 (61.5–80.5)	74.5 (68.0–82.0)	64.0 (45.3–76.5)	0.001
Octogenarians (*n*)	28 (30.4%)	22 (37.9%)	6 (17.6%)	0.041
Male (*n*)	47 (51.1%)	26 (44.8%)	21 (61.8%)	0.117
Height (cm)	159.3 ± 10.2	157.3 ± 10.3	162.7 ± 9.4	0.016
Body weight (kg)	60.0 ± 15.0	58.0 ± 13.3	63.2 ± 17.2	0.109
Body mass index (kg/m^2^)	23.4 ± 4.4	23.3 ± 3.8	23.7 ± 5.4	0.632
Hypertension (*n*)	64 (69.6%)	41 (70.7%)	23 (67.6%)	0.759
Hyperlipidemia (*n*)	27 (29.3%)	18 (31.0%)	9 (26.5%)	0.643
Diabetes mellitus (*n*)	4 (4.3%)	3 (5.2%)	1 (2.9%)	0.527
Current smoker (*n*)	26 (28.3%)	16 (61.5%)	10 (38.5%)	0.851
Chronic kidney disease (*n*)	16 (17.4%)	13 (22.4%)	3 (8.8%)	0.097
Serum creatinine (mg/dl)	0.91 (0.75–1.10)	0.83 (0.64–0.99)	1.03 (0.76–1.13)	0.340
History of percutaneous coronary intervention (*n*)	5 (5.4%)	2 (3.4%)	3 (8.8%)	0.261
COPD (*n*)	6 (6.5%)	5 (8.6%)	1 (2.9%)	0.275
Previous cardiac surgery (*n*)	4 (4.3%)	2 (3.4%)	2 (5.9%)	0.473
Malperfusion (*n*)	21 (22.8%)	9 (15.5%)	12 (35.3%)	0.029
Shock (*n*)	18 (19.6%)	14 (24.1%)	4 (11.8%)	0.149
Cardiac tamponade (*n*)	23 (25.0)	17 (29.3%)	6 (17.6%)	0.212
Impaired consciousness (*n*)	17 (18.5%)	12 (20.7%)	5 (14.7%)	0.475
Cardiopulmonary resuscitation (*n*)	7 (7.6%)	5 (8.6%)	2 (5.9%)	0.485
Predicted mortality by Japan SCORE (%)	9.0 (6.0–14.8)	9.5 (6.0–15.8)	8.3 (5.8–16.5)	0.560
Transfer distance (km)	50.9 (5.4–130.0)	49.7 (5.2–130.0)	52.3 (13.9–71.0)	0.758
Onset-to-arrival time (min)	221.0 (124.0–374.0)	228.5 (127.8–371.3)	220.0 (114.0–428.0)	0.866
Arrived within 6 h (*n*)	56 (60.9%)	39 (67.2%)	17 (50.0%)	0.102

Continuous variables are presented as means and standard deviations or medians and first and third quartile in cases of skewed data distributions. Categorical variables are presented as frequencies and percentages.

### Intraoperative data

Table 2 presents the intraoperative data. The intimal tearing sites were more likely to be confined within the ascending aorta in the PR group. In the ER group, the classical elephant trunk technique was performed for four patients (11.8%) and a FET was used for 26 patients (76.5%) in distal anastomosis. There were no significant differences between the two groups in terms of combined surgical procedures performed. Operative, cardiopulmonary bypass, aortic cross-clamping, and circulatory arrest times were significantly longer in the ER group. The amount of intraoperative bleeding and requirements for fresh frozen plasma and platelet concentrate were significantly greater in the ER group.

**Table 2:** Intraoperative data.

	Overall (*n* = 92)	Proximal repair group (*n* = 58)	Extensive repair group (*n* = 34)	*p*-value
**Intimal tearing sites**				
Ascending aorta (*n*)	40 (43.5%)	31 (53.4%)	9 (26.5%)	0.012
Beyond ascending aorta (*n*)	37 (40.2%)	19 (32.8%)	21 (61.8%)	0.057
Unidentified (*n*)	15 (16.3%)	8 (13.8%)	7 (20.9%)	0.394
**Combined procedures**				
Elephant trunk (*n*)		N/A	4 (11.8%)	
Frozen elephant trunk (*n*)		N/A	26 (76.5%)	
Aortic root replacement (*n*)	10 (10.9%)	4 (6.9%)	6 (17.6%)	0.110
Coronary artery bypass grafting (*n*)	5 (5.4%)	2 (3.4%)	3 (8.8%)	0.272
**Operative profiles**				
Operative time (min)	332.0 (290.3–433.3)	324.0 (289.0–351.3)	419.5 (326.5–553.0)	0.000
Cardiopulmonary bypass time (min)	155.0 (134.3–183.0)	152.0 (133.0–177.0)	170.0 (140.5–270.3)	0.001
Myocardial ischemia time (min)	96.5 (77.0–121.0)	94.0 (69.8–114.0)	109.5 (81.3–156.5)	0.002
Hypothermic circulatory arrest time (min)	37.0 (28.3–44.8)	35.5 (23.0–46.5)	41.0 (33.0–58.5)	0.012
Minimal rectal temperature (°C)	26.3 (25.4–27.7)	26.9 (25.9–27.8)	25.9 (24.9–26.7)	0.013
Bleeding amount (ml)	4,345.5 (2,533.8–7,571.8)	4,187.0 (2,660.5–7,132.5)	5,630.0 (2,832.8–11,858.3)	0.015
Red blood cells (U)	22.0 (16.0–29.5)	21.0 (15.5–28.5)	26.0 (22.0–31.5)	0.050
Frozen fresh plasma (U)	27.0 (20.0–36.0)	25.0 (20.0–36.0)	30.0 (25.0–40.0)	0.042
Platelet concentrate (U)	40.0 (40.0–60.0)	40.0 (38.7–55.0)	55.0 (40.0–60.0)	0.003

Continuous variables are presented as medians and first and third quartile. Categorical variables are presented as frequencies and percentages.

### Early outcomes

Table 3 shows the early operative outcomes. The overall operative mortality was 13.0%. There were no significant differences in terms of 30-day mortality and hospital mortality between the two groups. Ten patients (10.9%) experienced neurologic dysfunction, including five cases of stroke (5.4%) and five cases of temporary neurological deficit (5.4%). Spinal cord injury: paraparesis occurred in two cases (2.2%).

**Table 3:** Operative outcomes.

	Overall (*n* = 92)	Proximal repair group (*n* = 58)	Extensive repair group (*n* = 34)	*p*-value
**Early outcome**				
30 days mortality (*n*)	11 (12.0%)	6 (10.3%)	5 (14.7%)	0.379
Hospital mortality (*n*)	12 (13.0%)	7 (12.1%)	5 (14.7%)	0.475
Re-thoracotomy for bleeding (*n*)	7 (7.6%)	4 (6.9%)	3 (8.8%)	0.515
Mediastinitis (*n*)	3 (3.3%)	2 (3.4%)	1 (2.9%)	0.387
Temporary neurological deficit (*n*)	5 (5.4%)	4 (6.9%)	1 (2.9%)	0.387
Stroke (*n*)	5 (5.4%)	2 (3.4%)	3 (8.8%)	0.261
Paraparesis (*n*)	2 (2.2%)	1 (1.7%)	1 (2.9%)	0,605
Acute kidney injury (*n*)	16 (17.4%)	10 (17.2%)	6 (17.6%)	0.960
**Late outcomes**				
Follow-up (month)	29.0 (13.3–61.0)	23.5 (6.3–57.3)	32.5 (17.8–60.5)	0.719
Late death (*n*)	26 (28.3%)	18 (31.0%)	8 (23.5%)	0.440
Reintervention (*n*)	11 (12.0%)	4 (6.9%)	7 (20.6%)	0.055

Continuous variables are presented as medians and first and third quartile. Categorical variables are presented as frequencies and percentages.

### Late outcomes

**Figure 1:**
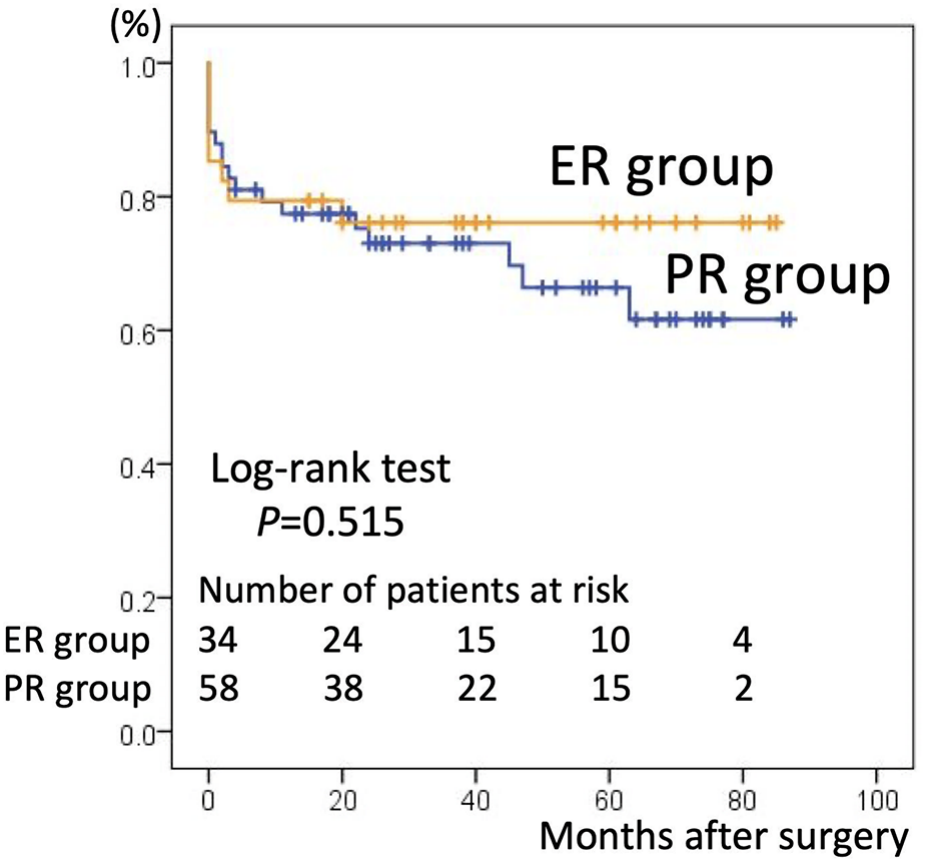
Kaplan–Meier curves for survival in the partial repair (PR) group and extensive repair (ER) group. The 5-year survival rate was 66.4% ± 7.1% in the PR group and 76.1% ± 7.4% in the ER group.

**Figure 2:**
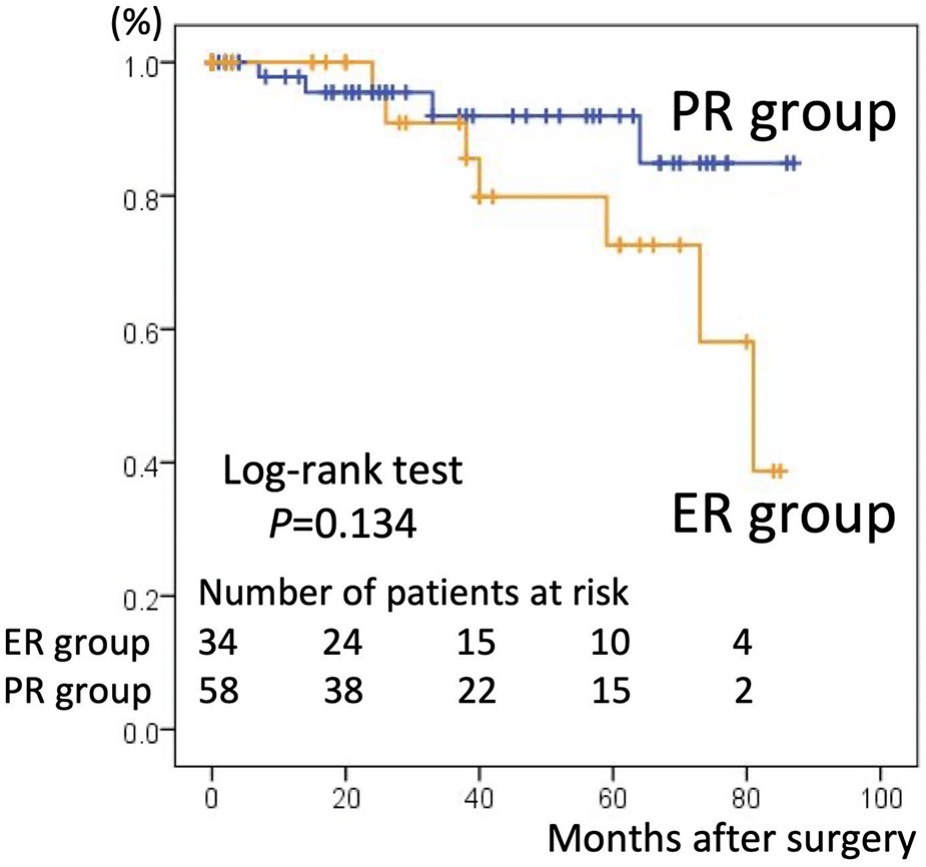
Kaplan–Meier curves for freedom from aortic reintervention in the partial repair (PR) group and extensive repair (ER) group. The 5-year freedom from aortic re-intervention rate was 92.9% ± 4.6% in the PR group and 72.6% ± 10.8% in the ER group.

There were 26 overall deaths during follow-up, 18 in the PR group and eight in the ER group (*p* = 0.440). The number of deaths after discharging from the hospital was 14. 11 patients in PR group were due to pulmonary (*n* = 4), intracranial bleed (*n* = 2), gastrointestinal (*n* = 2), cardiac (*n* = 1) and unknown cause (*n* = 2). 3 patients in ER group were due to mediastinitis (*n* = 1), gastrointestinal (*n* = 1) and stroke (*n* = 1). The estimated the 5-year survival rate based on Kaplan–Meier analysis was 66.4% ± 7.1% in the PR group vs. 76.1% ± 7.4% in the ER group, a difference that was not statistically significant (*p* = 0.515, Figure 1). The freedom from aortic reintervention rate at 5-year follow-up was 92.9% ± 4.6% in the PR group vs. 72.6% ± 10.8% in the ER group with close to significance (*p* = 0.134, Figure 2).

### Distal aortic events

The procedure of reintervention is listed in Table 4. During follow-up, four of 58 patients (6.9%) in the PR group required elective aortic reintervention for significant dilation of a patent false lumen, three patients underwent surgical arch replacement, and one patient underwent thoracic endovascular aortic repair (TEVAR) in the descending aorta. Seven of 34 patients (20.6%) in the ER group required aortic reintervention, including three patients who underwent TEVAR following TAR with FET. All reinterventions in both groups were performed safely, with no perioperative mortality observed.

**Table 4:** Aortic reintervention.

	Initial operation	Age	Interval (m)	Procedure of reintervention
Proximal repair group	Hemiarch replacement	78	12	Distal arch replacement
	Hemiarch replacement	73	4	TEVAR
	Hemiarch replacement	70	14	TAR + Bentall + CABG
	Hemiarch replacement	66	5	TAR + Bentall + CABG
Extensive repair group	TAR + FET + F - F bypass	69	53	Hemiarch replacement + AVR
	TAR + FET	56	11	TEVAR
	Partial arch + F - F bypass	85	0	BCT pseudoaneurysm resection
	TAR + FET	60	1	TEVAR
	TAR + FET	64	35	Asc.Ao pseudoaneurysm resection
	TAR	40	19	Lt. CCA-Lt.SCA bypass
	TAR + FET	43	1	TEVAR

TEVAR, thoracic endovascular aortic repair; TAR, total arch replacement; CABG, coronary artery bypass grafting; FET, frozen elephant trunk; F-F bypass, femoro-femoral bypass; AVR, aortic valve replacement; BCT, brachiocephalic trunk; Asc.Ao, Ascending aorta; CCA, common carotid artery; SCA, Subclavian artery.

## Discussion

The main finding of the present study was that there was no significant difference in the rate of long-term cumulative survival between the two surgical strategies. The rate of freedom from aortic reintervention was higher in the PR group than in the ER group with close to significance.

The present study showed that the 5-year survival rate between the two groups was not statistically different, though slightly lower in the PR than in the ER group (66.4% ± 7.1% vs. 76.1% ± 7.4%, *p* = 0.515). This result is in line with those of previous studies. Uchida et al. reported a higher survival rate in TAR at 5 years (69.0% in hemi-arch replacement vs. 95.3% in TAR, *p* = 0.03) (11), and Omura et al. reported favorable 5-year survival rates in discharged patients (83.8% in hemi-arch replacement vs. 88.6% in TAR, *p* = 0.54) (2). Other studies also showed that ER can be performed safely without increasing operative mortality and morbidity compared to PR (1, 4, 5, 7, 12, 13). However, Kim et al. (6) pointed out that the previous studies mentioned above (11–13) lack a control group and did not make adequate adjustment for baseline characteristics between the study and control groups. Therefore, more acceptable outcomes in the ER group may reflect a more favorable preoperative risk profile in the ER group compared with the PR group. After adjusting for multivariate variables, they reported that PR afforded better survival at 5 years than ER (83.2% vs. 65.8%, *p* = 0.01). The tendency for patients to have more favorable preoperative risk profiles in the ER group was also observed in the current study. The PR group was older and had a greater proportion of patients with preoperative shock, impaired consciousness, cardiac tamponade, or requiring CPR. At our institute, the surgical strategy was selected based not only on the location of the intimal tear, the range of dissection, the status of the true lumen, the diameter of aortic arch, and the age of the patient, but also on preoperative patient status. Therefore, with this unavoidable selection bias, preoperative patient status in the PR group was less favorable than that in the ER group and had more risk factors for mortality (such as need for CPR) (2, 14, 15). Thus, the lower long-term survival rate in the PR group could be explained by an unfavorable preoperative status.

The PR group had a higher free from reintervention rate than the ER strategy group (92.9% ± 4.6% vs. 72.6% ± 10.8%, *p* = 0.134), which has not been reported previously. Theoretically, incomplete resection of the dissected aortic arch may necessitate distal aortic reintervention (2, 12, 16). Typically, TEVAR is performed following hemiarch replacement because of progressive aortic dilatation. However, in our experience, although three cases of reintervention TEVAR were performed, the PR strategy did not correlate with an increased risk of future surgical reintervention. This result may be explained by two factors that were not uniform between the two groups: age and the location of the intimal tear. First, intimal tearing sites were more likely to be confined to the ascending aorta in the PR group. As complete resection of the intimal tear is the basis of treatment, this advantageous factor may explain the low rate of aortic reintervention in the PR group. Second, patients in the PR group were significantly older than those in the ER group. Considering the time to reintervention, a certain percentage of patients in the PR group may have died of other causes before experiencing progression requiring reintervention. PR can be performed less invasively than ER, and it usually allows a significant period without reintervention. As a result, PR is appropriate for elderly patients in circumstances requiring urgent life-saving intervention.

Conversely, ER was associated with a high risk of reintervention, which may be accounted for by the contents of late reintervention. As listed in Table 4, there were three patients who underwent TEVAR following TAR with FET. The FET technique is a concomitant procedure in TAR that was introduced more than a decade ago and has been widely adopted (13, 17, 18). It is generally accepted that FET can result in favorable aortic remodeling. However, it is also associated with the development of distal stent graft-induced new entry (dSINE) postoperatively (19). In all three cases, enlargement of the aneurysm diameter was observed, and these results were considered to be a consequence of dSINE. Although there was an aspect as a secured landing zone provided by FET may contribute for preferring TEVAR, dSINE provoked unwanted late reintervention. The other four cases of reintervention following TAR were all considered complications that were necessitated by a highly invasive and complex ER approach. As shown in Table 2, in comparison with PR, more operative time and a greater amount of human blood derivatives were required in ER. With the increased preference for more invasive surgical approaches, the incidence of postoperative complications requiring reoperation may be unavoidable.

Our institute services a large area in northern Hokkaido, covering an area of 18,000 km^2^ and populated by approximately 650,000 people. Because this area is large and has a low population density, long-distance transfers of patients with AADA to our institute are common. Although Andrew et al. (8) reported that interfacility transfer of patients with AADA, even with delay in surgery, did not affect the operative mortality rate; the desirable onset-to-operation time should theoretically be as short as possible. This is particularly important as the risk of death is estimated to be 1%–2% an hour (20). Caleb et al. showed that the time intervals between symptom onset, diagnosis, and surgery had a significant effect on mortality in patients with AADA (21), which supports the theory described above. As documented by the Japan Registry of Aortic Dissection (JRAD) (22), the median time from onset to arrival at hospital and the percentage of patients arriving within 6 h in Japan were 199 min and 67.4%, respectively, compared with 221 min and 60.9% in our study (Table 1). The percentage of octogenarians reported in the JRAD was 16.9%, compared with 30.4% in the current study. This suggests that a greater proportion of patients with a relatively unfavorable preoperative status are transferred to our institute.

Our study found that PR is less invasive than ER strategy and did not correlate with an increased risk of future surgical reintervention. In addition, reintervention in the PR group was performed electively. There was no perioperative mortality observed. Although the optimal extent of surgical resection and reconstruction of the arch in AADA is still under debate, and the surgical strategy should be selected taking into consideration multiple factors, our results suggest the adequacy of a limited approach for patients with a high risk profile, in preference for urgent life-saving therapy. In particular, in circumstances of relatively unfavorable preoperative patient status and a regional facility, limited aortic resection would be acceptable as not a palliative but a reasonable strategy.

### Study limitations

This study has several limitations. It was a retrospective analysis of our institutional database of prospectively collected observational data. Patient characteristics were not comparable between the study groups, and selection bias may exist regarding the choice of surgical strategy. Given the urgency of AADA, the potential for selection bias is unavoidable, and prospective randomization of surgical procedures would be impractical. Lower freedom from reintervention late in ER group was attributed partially to a dSINE following TAR with FET. In this respect, subsequent long-term follow-up are needed to confirm the result. Furthermore, our study was very limited by its small sample size. We believed it is worth revealing the “real-world” clinical outcomes in relatively small center, however, it is obvious that larger studies with longer follow-up periods are required to obtain more insights into this research area.

## Conclusions

No significant differences were found in the rates of long-term cumulative survival and freedom from aortic reintervention between the two surgical strategies. These findings support the theory that limited aortic resection achieves acceptable patient outcomes.

## Data Availability

The original contributions presented in the study are included in the article/Supplementary Material, further inquiries can be directed to the corresponding author.

## References

[1] Zhang H Lang X Lu F Song Z Wang J Han L Acute type A dissection without intimal tear in arch: proximal or extensive repair? J Thorac Cardiovasc Surg. (2014) 147(4):1251–5. 10.1016/j.jtcvs.2013.04.029 23778086

[2] Omura A Miyahara S Yamanaka K Sakamoto T Matsumori M Okada K Early and late outcomes of repaired acute DeBakey type I aortic dissection after graft replacement. J Thorac Cardiovasc Surg. (2016) 151(2):341–8. 10.1016/j.jtcvs.2015.03.068 26496808

[3] Poon SS Theologou T Harrington D Kuduvalli M Oo A Field M. Hemiarch versus total aortic arch replacement in acute type A dissection: a systematic review and meta-analysis. Ann Cardiothorac Surg. (2016) 5(3):156–73. 10.21037/acs.2016.05.06 27386403 PMC4893527

[4] Larsen M Trimarchi S Patel HJ Di Eusanio M Greason KL Peterson MD Extended versus limited arch replacement in acute type A aortic dissection. Eur J Cardiothorac Surg. (2017) 52(6):1104–10. 10.1093/ejcts/ezx214 28977503

[5] Ok YJ Kang SR Kim HJ Kim JB Choo SJ. Comparative outcomes of total arch versus hemiarch repair in acute DeBakey type I aortic dissection: the impact of 21 years of experience. Eur J Cardiothorac Surg. (2021) 60(4):967–75. 10.1093/ejcts/ezab189 33880505

[6] Kim JB Chung CH Moon DH Ha GJ Lee TY Jung SH Total arch repair versus hemiarch repair in the management of acute DeBakey type I aortic dissection. Eur J Cardiothorac Surg. (2011) 40(4):881–7. 10.1016/j.ejcts.2010.12.035 21315615

[7] Rylski B Beyersdorf F Kari FA Schlosser J Blanke P Siepe M. Acute type A aortic dissection extending beyond ascending aorta: limited or extensive distal repair. J Thorac Cardiovasc Surg. (2014) 148(3):949–54; discussion 954. 10.1016/j.jtcvs.2014.05.051 25018156

[8] Goldstone AB Chiu P Baiocchi M Lingala B Lee J Rigdon J Interfacility transfer of medicare beneficiaries with acute type A aortic dissection and regionalization of care in the United States. Circulation. (2019) 140(15):1239–50. 10.1161/CIRCULATIONAHA.118.038867 31589488 PMC9856243

[9] Qin W Su C Li L Carmichael M Huang F Chen X. Is limited aortic resection more justified in elderly patients with type A acute aortic dissection? Insights from single center experience. J Cardiothorac Surg. (2020) 15(1):183. 10.1186/s13019-020-01234-8 32703274 PMC7379362

[10] Nakanishi S Wakabayashi N Ise H Kitahara H Hirofuji A Ishikawa N Proximalized total arch replacement can be safely performed by trainee. Thorac Cardiovasc Surg. (2021) 69(4):336–44. 10.1055/s-0040-1713354 32634833 PMC8236320

[11] Uchida N Shibamura H Katayama A Shimada N Sutoh M Ishihara H. Operative strategy for acute type a aortic dissection: ascending aortic or hemiarch versus total arch replacement with frozen elephant trunk. Ann Thorac Surg. (2009) 87(3):773–7. 10.1016/j.athoracsur.2008.11.061 19231387

[12] Urbanski PP Siebel A Zacher M Hacker RW. Is extended aortic replacement in acute type A dissection justifiable? Ann Thorac Surg. (2003) 75(2):525–9. 10.1016/S0003-4975(02)04378-3 12607666

[13] Sun LZ Qi RD Chang Q Zhu JM Liu YM Yu CT Surgery for acute type A dissection using total arch replacement combined with stented elephant trunk implantation: experience with 107 patients. J Thorac Cardiovasc Surg. (2009) 138(6):1358–62. 10.1016/j.jtcvs.2009.04.017 19660407

[14] Goda M Imoto K Suzuki S Uchida K Yanagi H Yasuda S Risk analysis for hospital mortality in patients with acute type a aortic dissection. Ann Thorac Surg. (2010) 90(4):1246–50. 10.1016/j.athoracsur.2010.05.069 20868821

[15] Chiappini B Schepens M Tan E Dell’ Amore A Morshuis W Dossche K Early and late outcomes of acute type A aortic dissection: analysis of risk factors in 487 consecutive patients. Eur Heart J. (2005) 26(2):180–6. 10.1093/eurheartj/ehi024 15618075

[16] Suzuki T Asai T Kinoshita T. Predictors for late reoperation after surgical repair of acute type A aortic dissection. Ann Thorac Surg. (2018) 106(1):63–9. 10.1016/j.athoracsur.2018.01.071 29501645

[17] Tochii M Takami Y Ishikawa H Ishida M Higuchi Y Sakurai Y Aortic remodeling with frozen elephant trunk technique for Stanford type A aortic dissection using Japanese J-graft open stent graft. Heart Vessels. (2019) 34:307–15. 10.1007/s00380-018-1246-x 30191318 PMC6510868

[18] Roselli EE Idrees JJ Bakaeen FG Tong MZ Soltesz EG Mick S Evolution of simplified frozen elephant trunk repair for acute DeBakey type I dissection: midterm outcomes. Ann Thorac Surg. (2018) 105:749–55. 10.1016/j.athoracsur.2017.08.037 29217087

[19] Kreibich M Bünte D Berger T Vötsch A Rylski B Krombholz-Reindl P Distal stent graft–induced new entries after the frozen elephant trunk procedure. Ann Thorac Surg. (2020) 110:1271–9. 10.1016/j.athoracsur.2020.02.017 32194032

[20] Malaisrie SC Szeto WY Halas M Girardi LN Coselli JS Sundt TM 3rd, AATS Clinical practice standards committee: adult cardiac surgery. 2021 the American association for thoracic surgery expert consensus document: surgical treatment of acute type A aortic dissection. J Thorac Cardiovasc Surg. (2021) 162(3):735–758.e2. 10.1016/j.jtcvs.2021.04.053 34112502

[21] Matthews CR Madison M Timsina LR Namburi N Faiza Z Lee LS. Impact of time between diagnosis to treatment in acute type A aortic dissection. Sci Rep. (2021) 11(1):3519. 10.1038/s41598-021-83180-6 33568755 PMC7876041

[22] Inoue Y Matsuda H Uchida K Komiya T Koyama T Yoshino H Analysis of acute type A aortic dissection in Japan registry of aortic dissection (JRAD). Ann Thorac Surg. (2020) 110(3):790–8. 10.1016/j.athoracsur.2019.12.051 32035913

